# Tumour Microenvironment: The General Principles of Pathogenesis and Implications in Diffuse Large B Cell Lymphoma

**DOI:** 10.3390/cells13121057

**Published:** 2024-06-18

**Authors:** Stanislavs Sinkarevs, Boriss Strumfs, Svetlana Volkova, Ilze Strumfa

**Affiliations:** Department of Pathology, Riga Stradins University, 16 Dzirciema Street, LV-1007 Riga, Latvia

**Keywords:** diffuse large B cell lymphoma (DLBCL), tumour microenvironment (TME), tumour-associated macrophages (TAM), tumour-associated neutrophils (TAN), T cell, cancer-associated fibroblasts (CAF)

## Abstract

Diffuse large B cell lymphoma (DLBCL) is the most common type of non-Hodgkin lymphoma worldwide, constituting around 30–40% of all cases. Almost 60% of patients develop relapse of refractory DLBCL. Among the reasons for the therapy failure, tumour microenvironment (TME) components could be involved, including tumour-associated macrophages (TAMs), myeloid-derived suppressor cells (MDSCs), tumour-associated neutrophils (TANs), cancer-associated fibroblasts (CAFs), and different subtypes of cytotoxic CD8+ cells and T regulatory cells, which show complex interactions with tumour cells. Understanding of the TME can provide new therapeutic options for patients with DLBCL and improve their prognosis and overall survival. This review provides essentials of the latest understanding of tumour microenvironment elements and discusses their role in tumour progression and immune suppression mechanisms which result in poor prognosis for patients with DLBCL. In addition, we point out important markers for the diagnostic purposes and highlight novel therapeutic targets.

## 1. Introduction

Diffuse large B cell lymphoma (DLBCL) is the most common type of non-Hodgkin lymphoma worldwide, constituting around 30–40% of all cases in different geographic regions [1]. According to the Munich Cancer Registry, collecting data from the Upper-Bavarian region of Germany, around 211 cases of DLBCL were registered per year, corresponding to an incidence of 4.2/100,000 [2]. Based on United States cancer registry data, the incidence of DLBCL is 7.2/100,000 [3]. The incidence increases with age and generally is higher in males than in females [2,3]. The 5-year survival in DLBCL patients, according to National Cancer Institute (NIH) data for the time period 2014–2020, is 64.7% with mortality 1.7/100,000 [4]. In the Upper-Bavarian region of Germany, mortality constitutes around 43.7% for the same period [2]. A significant impact on the survival was reached with the invention of anti-CD20 monoclonal antibodies such as Rituximab, which boosted the complete response rate to 76% with the R-CHOP treatment scheme (compared to 63% with the standard CHOP protocol) [5]. Approximately 30% to 40% of patients with DLBCL achieve and maintain complete remission after first-line therapy. New challenges appear along the course of treatment, such as so-called relapse of refractory DLBCL (RR-DLBCL) [6]. According to the SHOLAR-1 study group, which aims to improve treatment options of RR-DLBCL, only 26% of patients had a response to the next line of therapy, with a median survival rate of 6.3 months. Only 20% of patients with refractory disease were alive at 2 years [7]. The poor outcomes have moved the focus of modern studies towards better understanding of DBLCL and its microenvironment (TME), which play a significant role in the development of resistance against the main treatment scheme.

The aim of this review is to provide comprehensive pathogenetic analysis of the components of the DLBCL microenvironment in the wider context of carcinogenesis and to make a correlation between TME and future treatment options in order to improve our understanding of DLBCL and its treatment options.

## 2. Components of Diffuse Large B-Cell Lymphoma

The interaction between tumour cells and the nearby peritumoural stroma has been thoroughly investigated in numerous cancer types over the past few decades as a dynamic system that encompasses carcinogenesis, tumour invasion, and metastatic spread. The tumour components are classified as cellular ones, including the neoplastic cells; fibroblasts; immune, endothelial, mesenchymal stem cells; and non-cellular compounds, including growth factors, cytokines, extracellular matrix, hormones, and even viruses. All the non-neoplastic components create the tumour microenvironment (TME) [8]. In the modern WHO classification (2022), DLBCLs are classified by cell-of-origin (COO) classification based on gene-expression profiling: the activated B-cell-like (ABC) and the germinal centre B-cell-like (GCB) and not-yet classified type 3 or T cell/histiocyte-rich large B-cell lymphoma (T/HRLBCLs) [9,10]. Activated B-cell type shows significantly worse prognosis and higher resistance to the standard R-CHOP regimens. Double-hit variants which overexpress MYC and BCL2 proteins are defined as aggressive DLBCLs and are also associated with a poor prognosis [11]. Looking into cellular TME, many studies report on infiltration of tumour-associated macrophages (TAMs), myeloid-derived suppressor cells (MDSCs), tumour-associated neutrophils (TANs), natural killer cells (NKc), cancer-associated fibroblasts (CAFs), and dendritic cells (DCs) [12,13,14,15,16,17]. These cellular components may exhibit pro- and/or anti-tumourigenic functions affecting the prognosis as well become targets for additional therapy (Table 1).

## 3. Tumour-Associated Macrophages (TAMs)

**Figure 1:**
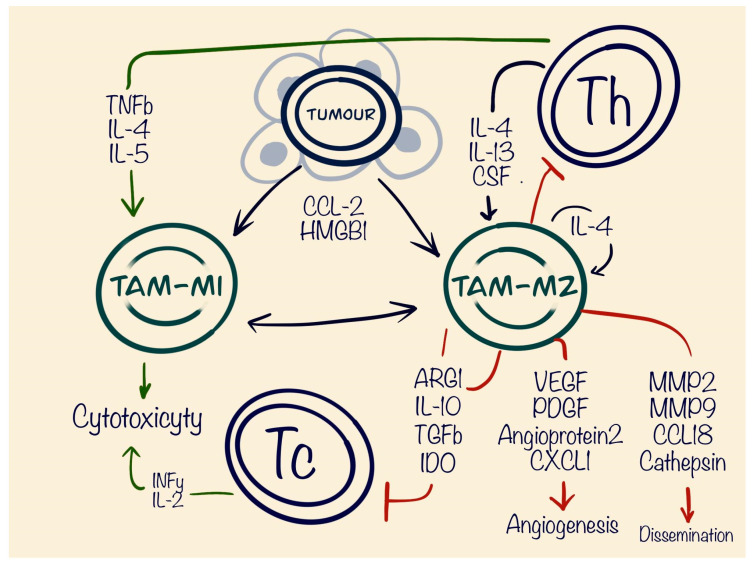
Role of tumour-associated macrophages (TAM) in tumour microenvironment (TME). Figure shows pro-tumourous effects of TAM-M2 cells as suppressing of cytotoxic and helper T cells, stimulating angiogenesis and dissemination of the tumour. The TAM-M1 type has an anti-tumourous function by cytotoxicity and phagocytosis.

Tumour-associated macrophages (TAMs), a prominent type of tumour-infiltrating immune cells, are typically classified into two functionally distinct subtypes. The first subtype is represented by classically activated (M1) macrophages, which have proinflammatory and anti-tumour properties, allowing them to precisely identify and eliminate cancer cells through cytotoxicity and phagocytosis. On the other hand, alternatively activated (M2) macrophages exhibit anti-inflammatory characteristics and play a role in tissue repair and growth, which works as a pro-tumourous factor, making a more “comfortable” environment for tumour cells [18]. Importantly, TAMs of both M1 and M2 types can dynamically transition between phenotypes in response to changes within the tumour microenvironment. The polarisation of TAMs is influenced by a diverse array of cytokines, growth factors, chemokines, and other signals emanating from tumour and stromal cells. CCL2 is the primary chemokine expressed by tumour cells and plays a key role in attracting immune cells, particularly TAMs, via the CCL2/CCR2 axis [19]. When the CCR2 receptor is activated by its ligand CCL2, it initiates diverse G protein-mediated signalling cascades inside the cell, including the phosphatidylinositol 3-kinase (PI3K)/AKT, mitogen-activated protein kinase (MAPK)/p38, and Janus kinase (JAK)/ transducer and activators of transcription 3 (STAT3) pathways. The activation of these signalling pathways is crucial for anti-apoptosis, angiogenesis, and cell migration, which collectively contribute to oncogenic progression [20]. Polarisation towards the M2 phenotype is usually induced by the presence of Th2 cells producing interleukins 13 (IL-13) and 4 (IL-4), as well as enhanced expression of colony-stimulating factor 1 receptor (CSF1R) on the surface of a macrophage [21]. Functionally, these macrophages facilitate tumour immune evasion, promote angiogenesis, and contribute to tumour growth and dissemination [22]. M2-like macrophages secrete factors including vascular endothelial growth factor (VEGF), platelet-derived growth factor (PDGF), angiopoietin 2, CXCL1, and fibroblast growth factor 2 (FGF-2) for stimulating angiogenesis [23]. Matrix metalloproteinase 2 (MMP2) and 9 (MMP9), cathepsin, CCL18, and CYP4A promote extracellular matrix (ECM) breakdown and stimulate tumour dissemination. The third important function of the TAMs is interaction and suppression of T cells via arginase 1 (Arg-1), IL-10, transforming growth factor beta (TGF-β), and indolamine 2,3-dioxygenase (IDO), resulting in local immune response evasion [24]. TAMs have been shown to produce cytokines such as C5a, IL-6, and TNF-α, which activate the STAT3 and nuclear factor-κB (NF-κB) pathways, that are important for the macrophage polarisation. The process can be directed towards the M1 phenotype through STAT1 signalling with the help of adaptor proteins MyD88 and TRIF, which regulate signalling downstream of TLR4, an important activator of NF-κB and, on the opposite side, the M2 phenotype is promoted through STAT6 [25] signalling induced by IL-4 and IL-13 [26]. Huber et al., analysing the TME of melanoma, found an increased level of High-Mobility Group Box 1 protein (HMGB1) levels in patients’ serum, compared to the healthy patients, secreted by the melanoma tumour. HMGB1 had a direct impact on recruitment of M2 macrophages and production of interleukin 10 (IL-10) through a receptor for advanced glycation end product (RAGE)-dependent signalling [27]. IL-10 has an ability to suppress the production of IL-2 and interferon-γ (IFN-γ). Additionally, it can affect CD4+ T cells by suppressing their antigen-specific activation and proliferation in lymph nodes, reducing their release of cytokines like IL-4, IL-5, and TNF-α, as well as their cytotoxic capabilities [28], which are crucial for the activation of M1 macrophages and anti-tumourous effects, as well producing IL-4 and host a positive feedback loops promoting more M2 macrophages [29] (Figure 1).

To have a potential treatment aimed at TAM M2 cells, it is crucial to identify their presence in the tumourous tissues. Significant markers which are specific for M2 cells are mannose receptor (CD206) and scavenger receptor hemoglobulin (CD163). These receptors could be potentially diagnosed by immunohistochemistry or flow cytometry methods [30]. Potential targeting of TAMs could be beneficial in complex use along with other regimens. Among the current research focuses, there is the approach to inhibit TAM recruitment by blocking CSF-1R with monoclonal antibodies such as Emactuzumab (RG7155) [31,32] or tyrosine kinase inhibitor Pexidartinib (PLX3397). Another promising approach would be to block CCR2 receptors. Some studies have already shown improved overall survival compared to standard regimens (29% versus 18.6%) by using CCR2 inhibitor CCX872 in advanced pancreatic cancer [33]. Potential targeting of CCL2 with human monoclonal antibody Carlumab also may show beneficial results in the future [30].

In DLBCL the prognostic importance of the total population of CD68+ macrophages and even M2 macrophages is less clear. The published data have revealed that bulky mass and a higher number of M2 TAMs were significant factors for poor prognosis (*p* < 0.05) [34]. However, in another report, a high presence of TAMs showed association with more favourable prognosis, increased 5-year progression-free survival and overall survival. In patients who were treated with chemotherapy (CHOP regimen), high expression of the CD68+ cells revealed poor prognosis, but addition of rituximab to chemotherapy reversed the negative prognostic impact of high CD68+ TAM content to favourable [35]. In addition, there are publications where no associations between CD68+ TAMs and other clinical factors or prognostic outcome in DLBCL patients were reported [36]. J. E. Chang et al. have reported that a combination of R-CHOP regiments, together with GM-CSF, increases overall survival in patients with DLBCL [37]. GM-CSF was found to contribute to PD-L1 overexpression through the activation of the STAT3 pathway, alongside interferon (IFN)-γ [38] and induce repolarisation of M2 macrophages to M1 based on the PI3K/AKT/mTOR pathway [25]. The importance of TAMs is crucial in the TME; therefore, more clinical studies of targeting macrophages are needed to find novel treatment options for patients affected by DLBCL, and especially refractory DLBCL.

## 4. Myeloid-Derived Suppressor Cells (MDSCs)

The story of the myeloid-derived suppressor cells (MDSCs) started to evolve in 1978. In tumour-bearing mice models, immature myeloid cells were found to have the potential of suppressing T cell function and proliferation and thus consequently impacting poor prognosis. In further studies, researchers started to use an alternative name, myeloid suppressor cell, based on the cell role in the tumour and its inflammatory microenvironment [39]. Because this nomenclature was misleading and could result in diverse misunderstandings, Dmitry I. Gabrilovich et. al. suggested the term myeloid-derived suppressor cells, that combines the nature/origin and the function of the observed cells [40]. MDSCs originate from common myeloid progenitor cells in the bone marrow (BM). Their development is regulated by a complex array of signals, broadly categorised into those that encourage the accumulation of immature myeloid cells and those that lead to the pathological activation of these cells. Many studies have observed the accumulation of MDSCs in the BM of tumour-affected hosts, highlighting the changes in the myeloid compartment due to the presence of cancer in the body [41,42]. The pathological activation of MDSCs results from ongoing, relatively weak stimulation from tumour-derived signals in contrast with the fast and strong activation of myeloid cells by infections such as bacteria or viruses, which involves rapid differentiation into mature cells [43]. Stimulation of MDSCs in cancer patients is mostly driven by tumour-derived growth factors such as granulocyte-macrophage colony-stimulating factor (GM-CSF), granulocyte colony-stimulating factor (G-CSF), macrophage colony-stimulating factor (M-CSF), VEGF, and IL-6 [44,45]. These growth factors co-function through the signals via transducer and activators of transcription 1 (STAT1) [46], STAT3 [47] and STAT6 as well as retinoblastoma protein 1 (RB1) [48].

**Figure 2:**
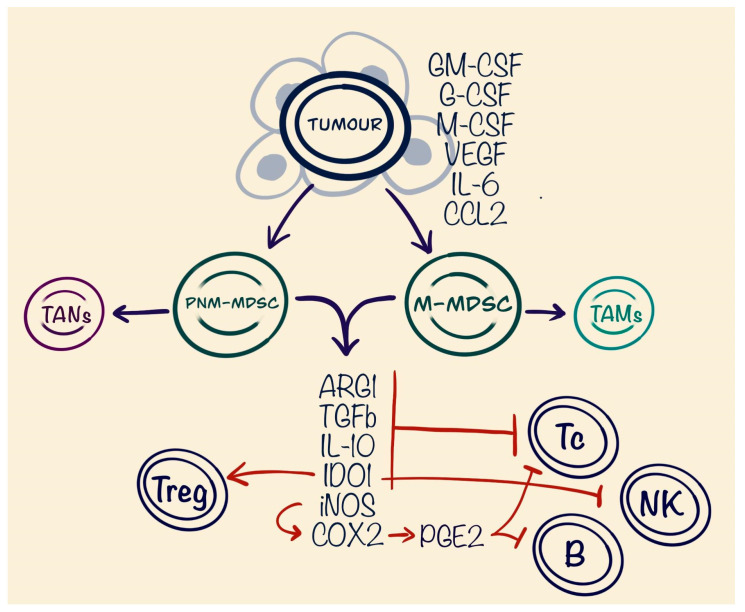
Role of myeloid-derived suppressor cells (MDSCs) in tumour microenvironment (TME). Figure shows negative effects mostly aimed at immune suppression in the TME.

MDSCs are composed of two major categories of cells, known as granulocytic or polymorphonuclear (PMN-MDSCs), which share phenotypic and morphological characteristics with neutrophils, and monocytic (M-MDSCs), which are similar in phenotype and morphology to monocytes [49]. MDSCs have a phenotype of CD11b+ and CD33+. In addition to this, PMN-MDSCs also are CD15+ and CD66b+ compared to the M-MDSCc, which are CD14+ [50]. The main factors in MDSCs immune-suppressing abilities involve arginase 1 (ARG1), iNOS, TGF-β, IL10, cyclooxygenase-2 (COX-2), and indoleamine 2,3-dioxygenase (IDO) expression on T cells. Increased ARG1 expression has been found in many tumours, e.g., hepatocellular carcinoma, non-small cell lung cancer, and carcinoma of the large bowel. Increased synthesis of ARG1 by MDSCs strongly correlated with an increased risk of relapse in breast carcinoma [51]. ARG1 catabolises arginine, an amino acid required for T cell activation and proliferation [52]. NO products result in increased Nox2 activity and production of prostaglandin E2 (PGE2). PGE2 inhibits signalling via T cell receptors, potentially contributing to the resolution of inflammation. Furthermore, PGE2 restricts the immune response by blocking B-lymphocyte differentiation and impairing their capacity to present antigens [53]. TGF-β-deficient mice models have shown reduced proliferation of T cells through several mechanisms, including modulation of the mechanistic target of rapamycin (mTOR) and Forkhead box O3 (FOXO3) [54]. The mechanism by which IDO1 induces immunosuppression remains partially unclear, yet it is established that elevated levels of IDO1 can suppress natural killer (NK) cell activity, inhibit effector T cell activation, and promote the activation of regulatory T cells (Tregs) as well as the differentiation of tolerogenic dendritic cells. Additionally, IDO1 fosters the growth and activation of myeloid-derived suppressor cells. It also blocks the target of rapamycin complex 1 (mTORC1), which leads to T cell apoptosis and reduces inflammation mediated by antigen-presenting cells [55] (Figure 2).

Many studies have reported on the presence of MDSCs as a poor prognostic factor in diverse types of tumours, including Hodgkin’s and B cell lymphomas, gastric cancer, and colorectal carcinoma, thus highlighting MDSC as potential targets in the treatment of haematological and solid malignant tumours [56,57,58,59]. Treatment options targeting MDSCs may be oriented toward depletion and differentiation by using standard chemotherapy regimens such as 5-fluorouracil, carboplatin, or paclitaxel, and showed increased levels of INF-y, which was crucial for development of cytotoxic CD8+ T cells, but unfortunately worked as a double-edged sword by deficiency of specificity on the MDSCs [59]. Better targeted therapy could be based on the CD33 monoclonal antibodies such as gemtuzumab ozogamicin (GO), which is an approved treatment regimen for acute myeloid leukaemia [60]. There is still a lack of clinical trials of use of GO in targeting TME, but it could be a promising future treatment option. Targeting of MDSCs function could be a good additional component of complex treatment improving the prognosis of the patients. Meyer et al. have reported on benefits of phosphodiesterase-5 (PDE5) inhibitor sildenafil to impact MDSCs function by decreasing secretion of ARG1, IL-1β, IL-6, and VEGF in tumour-bearing mice and thus improving their survival [61]. Additionally, COX-2 inhibitors may play a significant role by blocking PGE2 production and, through that, inhibit MDSCs and increase T cell activity. The last target option is to disrupt chemotaxis of MDSCs into the tumour niche. STAT3 inhibitors nowadays are considered a potential addition to the immune therapy. The absence of crucial immunosuppressive factors in the TME may improve the outcomes in patients with metastatic or refractory diseases [62]. In DLBCL, the quantity of M-MDSCs has been linked with the International Prognostic Index, event-free survival, and the number of circulating Tregs. Additionally, depletion of monocytes resulted in restored T-cell proliferation. The suppression of T cells by myeloid cells was associated with the release of interleukin-10 and S100A12, along with an increase in PD-L1 expression [63]. Similar findings were observed in anaplastic DLBCL, where higher levels of MDSCs were associated with increased PD-L1 expression [64]. The most important idea of targeting MDSCs is increased activity and the presence of cytotoxic T cells in the tumour niche, which could increase the efficiency of the immune therapy and targeting PD-1/PD-1L with drugs such as pembrolizumab.

## 5. Tumour-Associated Neutrophils (TANs)

**Figure 3:**
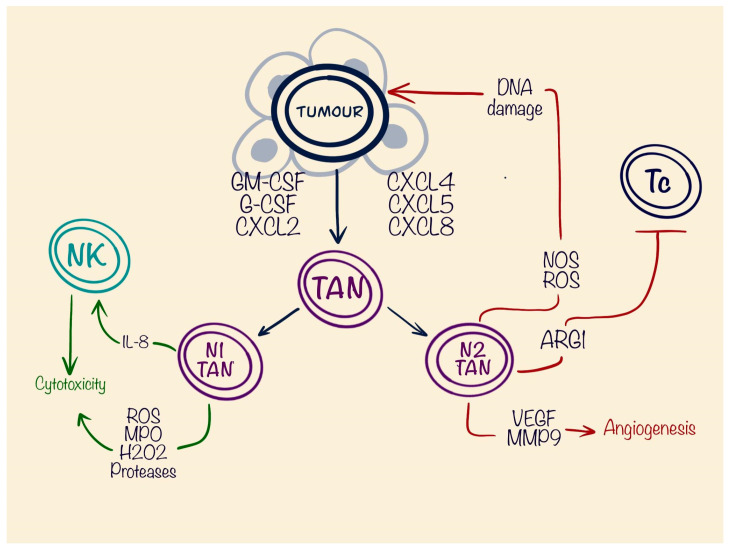
Role of tumour-associated neutrophils (TANs) in the tumour microenvironment. N1-TANs have shown cytotoxicity by producing toxic granules and have anti-tumourous effects. N2-TANs, conversely, are pro-tumourous, by increasing DNA damage to the tumour, which leads to the new mutations in the DNA, suppressing cytotoxic T cells and stimulating angiogenesis.

Neutrophils are the largest group of immune/inflammatory cells in the peripheral blood, constituting around 50–70% of all leukocytes. These short-living cells, having a life span around 7 h, are well known for their active response in acute inflammation scenarios [65]. Only recently, their role has started to be assessed from the perspectives of chronic inflammatory response and especially in the pathogenesis of the tumour microenvironment. In solid tumours, an abundance of neutrophils often correlates with poor outcome and prognosis, as reported in melanomas [66], bronchoalveolar carcinomas [67], or head and neck squamous cell carcinomas [68]. Neutrophils in cancer patients exert a pro-tumoural or an anti-tumoural effect [16], which make their role ambiguous in the TME. Neutrophils, similarly to the PMN-MDSCs, take their origin from multipotent granulocyte–monocyte progenitors (GMPs) [69] and are stimulated by GM-CSF and G-CSF [70], by which they share a similar CD14−CD15+CD66b+CD16+ phenotype [65,71]. Recruitment of the TANs is mediated through different chemokine receptors CXC-chemokine receptor 2 (CXCR2), CXCR4, and CXCR5 and their ligands. Expression of CXC-chemokine ligand 12 (CXCL12) by bone marrow stromal cells is responsible for retaining CXCR4+ immature neutrophils [72]. In hepatocellular carcinoma models, CXCR2 is activated via Erk1/2, p38, and NF-κB signals, and increases the life-span of the neutrophils together with pro-tumour effects. [73]. Polarisation of the TANs also plays a crucial role in tumourigenesis. Significant impact on differentiation into the TANs N2 type or pro-tumourous TANs develops through the TGF-β cytokine. This also works negatively on the differentiation towards anti-tumourous or N1 type. A completely opposite process is involved in the appearance of N1-TANs, where the crucial molecule is INF-β [74]. This fact shows plasticity of the TANs depending on the spectrum of secreted cytokines by tumour and surrounding tissues. Anti-tumourous effects are mostly based on the release of toxic granules, containing reactive oxygen species (ROS), myeloperoxidase (MPO), peroxides, and proteases. These compounds are highly effective in antimicrobial protection, but also have a potential for anti-tumour activity [73]. Except the granules, N1 TANs produce cytokines such as IL-8, which promotes proliferation of the NK-cells into the tumour niche and indirectly promotes cytotoxic immune response [75] (Figure 3).

Some authors have noted infiltration of TANs as favourable for overall survival (OS) in non-small cell lung cancer [76], gastric cancer, and oesophageal cancer [77]. On the other hand, N2 TANs may play a significant role in the tumourigenesis, by producing nitric oxide synthase (NOS). Multiple studies concluded that the number of neutrophils strongly correlated with the mutational burden in the tumour, as NOS and ROS, the most secreted molecules of neutrophils, have the potential to damage DNA [78,79,80]. Genetic instability stimulates tumour proliferation and survival despite the standard therapy [81]. This mechanism is completely opposite to the cytotoxic effects of ROS. Evidently, the result is dependent on the concentrations of ROS molecules in the tumour niche as well as the type of neutrophils. This process is most probably controlled by the tumour, and more studies are necessary to predict the role of the TANs in the TME. Another pro-tumourous molecule secreted by the TANs is ARG1 [82], which, in the same manner as MDSCs, suppress cytotoxic T cell proliferation. Production of the VEGF and matrix metalloproteinase-9 (MMP-9) also stimulates tumour angiogenesis. The amount of MMP-9 produced by the TANs is much higher compared with TAMs [83], which may indicate worse prognosis and higher risk of metastatic spread of the tumours. In therapeutic aims, TANs are still controversial cells, because of their short life-span and controversial function. Even as a prognostic marker, TANs are hard to use practically, because of the controversial reports in different studies. Still, more research is needed to understand TANs’ role in the tumour TME. In DLBCL, the pro-tumourigenic activities of neutrophils are believed to dominate, contributing to a poor prognosis. Notably, the CXCL-8/CXCR-1 pathway was identified as a significant target, presenting a potential therapeutic strategy for addressing tumour-associated neutrophils [16,84].

## 6. Cancer-Associated Fibroblasts (CAFs)

Cancer-associated fibroblasts are an important component in the tumour, constituting and building the supportive skeleton of the tumour—the stroma. CAFs represent the “soil” of the malignancy and secrete growth factors, inflammatory ligands, and extracellular matrix proteins that encourage tumour proliferation, therapy resistance, and immune exclusion, leading to poor prognosis and shorter overall survival [85]. CAFs are a heterogenous population of cells; therefore, identification and isolation of specific types can be difficult. One of the differentiation studies was done by immunohistochemistry on two major CAF groups: alfa smooth muscle actin (aSMA) high and IL-6 low group, better known as myofibroblasts (myCAFs), and aSMA low and IL-6 high or inflammatory CAFs (iCAFs). myCAFs usually are driven by the tumour and are located near tumour cells, promoting dual functions such as restricting or promoting tumour growth depending on the tumour stage [86]. iCAFs, driven by secreted IL-1 and TNF-α and located more distantly from the tumour, are generally confirmed as tumour-promoting cells through secretion of inflammatory cytokines and growth factors, stimulating proliferation, metastasis, and chemoresistance. CAFs are not permanent and can shift back and forth between subtypes using the TGF-β signalling pathway [87]. Another subtyping approach was suggested by Cords et al. using scRNA-seq to identify matrix CAFs (mCAFs), associated with upregulated expression of matrix metalloproteinase 11 (*MMP11*) and collagen 1A2 (*COL1A2*) genes. The importance of mCAFs is associated with the matrix remodelling features and formation of the stroma. The second-largest cluster was identified as inflammatory CAFs (iCAFs) expressing the *PLA2G2A* gene as well as showing association with complement system genes and CD34 [88]. iCAFs are promoted by fibroblast activation protein (FAP) and STAT3 activation, resulting in secretion of CCL2 and IL-6, significant for promoting MDSCs infiltration into the tumour microenvironment and supporting the growth of malignant cells [89]. The third important group of CAFs was named vascular cancer-associated fibroblasts (vCAFs). They overexpress *NOTCH3*, *COL18A1*, and *MCAM*, significant for vascularisation of the tumour. Another group of fibroblasts were found in tumour-derived hypoxic regions and secrete membrane metalloprotease (MME) TMEM158 and hypoxia marker carbonic anhydrase IX (CAIX), an indicator of the RAS pathway activation and promoting angiogenesis. Because of the similarity to the tumour behaviour, this cluster was named tumour CAFs (tCAFs) [88]. Significant markers, which may help identify CAFs, are αSMA, PDGFRα, and FAP. A significant disadvantage lies in the heterogeneity of CAFs and the corresponding lack of marker specificity [90], which make CAFs a challenge for targeted therapy. In the mice model of pancreatic ductal adenocarcinoma (PDA), Feig et al. noticed ineffectiveness of checkpoint inhibitors targeting anti-cytotoxic T-lymphocyte-associated protein 4 (α-CTLA-4) and α-programmed cell death 1 ligand 1 (PD1-L1). PDA had large infiltration of FAP+ CAFs overexpressing CXCL12 and the absence of cytotoxic T cells. After administration of AMD3100, a CXCL12 inhibitor, it turned over the response to checkpoint inhibitors and dramatically increased the presence of T lymphocytes, proving the anti-inflammatory potential of CAFs, resulting in the resistance to therapy [91]. Analysis of gastric cancer has shown that high-CAF groups have a positive correlation with M2-TAMs and M-MDSC and result in pro-tumourous effects and poor prognosis. The low-CAF group, oppositely, showed increased numbers of CD4+ T cells (Th1 and Th2) and correlated with better prognosis due to anti-tumourous function of T cells [92]. Hegab et al. reported on CAF presence in mice bearing lung adenocarcinoma. Tumours showed dependence on fibroblast growth factor (FGF), and production of FGF9 from CAFs resulted in multiple adenocarcinoma-like tumour nodules. Also, the model showed increased levels of TAMs and transforming growth factor beta (TGF-β), MMP7, FGF9, and FGF2 in the tumour niche. In vivo inhibition of FGF9 resulted in fewer tumour nodules [93]. Multiple studies have reported overexpression of TGF-β in high-CAF groups [87,88,93,94]. High levels of TGF-β may predict the resistance to the checkpoint inhibitors by suppressing T cell proliferation in TME [95], which makes TGF-β a potential target as an addition to the checkpoint inhibitor therapy [96]. Administering both TGF-β inhibitor SRK-181-mIgG1 and an anti-PD-1 antibody to mice with tumours that were resistant to anti-PD-1 therapy resulted in significant tumour reduction and increased survival rates [97]. Additionally, it boosted the effectiveness of 5-fluoruracil (5-FU) in the invasive behaviour of colorectal cancer cells by increasing E-cadherin levels and suppressing the enzymatic activity of MMP-9 [98], suppressing c-Myc expression in osteosarcoma cells, enhancing immune effectors (IFNγ+CD8+ cells and NK cells), and reducing the number of immune suppressors (M2-like TAMs, MDSCs) in the tumour microenvironment [99]. In DLBCL, Kotlov et al. noted an association between TAMs and CAFs, leading to the calculation of a TAM/CAF ratio. This ratio correlated with an increased risk of mortality. Furthermore, in the segment of the DLBCL matrisome with a lower TAM/CAF ratio, there was an enrichment of CAF-associated proteins, which was associated with a more favourable prognosis [84].

## 7. Role of T Lymphocytes in Tumour Pathogenesis

**Figure 4:**
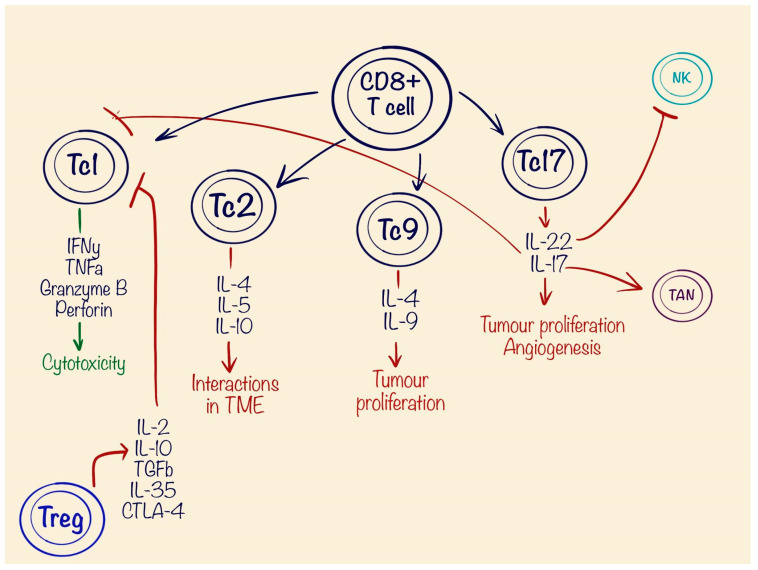
Different types of CD8+ T cells (Tc): Tc1, Tc2, Tc9, and Tc17 and their potential role on the tumour microenvironment (TME). Also illustrated is the immune-suppressing function of the T regulatory (Treg) cells.

T lymphocytes are the main regulators of immune response in the tumour microenvironment. There are two main types of T cells: CD4+ cells, better known as T helper (Th) cells, which work as coordinators of immune cells, and CD8+, which are known for their cytotoxic abilities (Tc), precisely targeting tumour cells [100]. The modern anticancer therapies focus on the cytotoxic abilities of T cells and potential activation of them in the tumour domain, and consist of checkpoint inhibitors such as PD-1 inhibitors like Pembrolizumab [101], chimeric antigen receptor T (CAR-T) cell therapy [102], bispecific antibodies (BsAbs) [103], and so-called nanobody complexes [104]. The effectiveness of these technologies can be boosted with a higher presence of the Tc in tumour tissues. CD8+ T cells are often considered a homogeneous group of cells known for secreting significant quantities of IFN-γ, TNF-α, the protease granzyme B and perforin, but recent studies have identified multiple subtypes of Tc, which reflect the spectrum of CD4+ T cells and are upregulated by different interleukins. There are four main subtypes: Tc1s, Tc2s, Tc9s, and Tc17s [105]. The Tc1 type mostly corresponds to classical CD8+ cells, which are promoted mostly by IL-12, secreted from Th1 [105,106,107]. Tc1s secrete IFN-γ and TNF-α and feature significant anti-tumour effects causing cell lysis. The Tc2 type, which secretes IL-4, IL-5, and IL-10, lack production of INF-γ and, by that, yield minor or no effect on cytotoxicity [108]. The presence of IL-4 plus TGF-β develops unique type CD8+ cells such as Tc9, which are special due to producing high amounts of IL-9 [109]. Tc9 lacks granzyme B, which results in weak cytotoxic activity. Moreover, Tc9 shares similar activation pathways and regulation mechanisms as Th9, which makes them closer to the regulatory function during inflammatory response [105]. The importance of IL-9 in tumour progression is controversial. On one hand, IL-9 correlates with lower amounts of IL-4, IL-10, VEGF, and TGF-β, which are important for tumour growth. In multiple models, IL-9 showed an association with improved prognosis and overall survival, when secreted [110]. On the other hand, other studies showed the involvement of IL-9 in the pathogenesis of multiple malignancies, such as lymphoma, leukaemia, and lung, breast, and thyroid cancers. IL-9 promotes cell proliferation and protects cancer cells from apoptosis by downregulating the JAK/STAT pathway [111] (Figure 4).

In haematological malignancies, IL-9 could play a special role, as it is important for lymphocyte activation and proliferation. In DLBCL, IL-9 promotes cell survival and drug resistance by upregulating *p21CIP1* genes. Also, DLBCL showed increased expression of the IL9 receptor on the cell surface and was associated with poor prognosis [112]. IL-9 also shows a significant role in chronic lymphocytic leukaemia, Hodgkin lymphomas and cutaneous T cell lymphomas [110]. Another subtype, known as Tc17, also has shown its correlation with inflammatory response in tumours. Their differentiation from CD8+ naive cells is induced by IL-6 or IL-21 along with TGF-β. Similarly to the Tc9, they exhibit reduced production of the INF-γ and granzyme B and produce IL-17 and IL-22 [105]. Akbay et al., in a study with a mice model of lung cancer, concluded that excessive production of IL-17 had a direct correlation with cancer growth and infiltration of TANs into the tumour TME, and reduced the number of cytotoxic lymphocytes, thus increasing resistance to the PD-1 inhibitors [113]. A study on uterine cervical cancer showed an association of Tc17 promoting Th17 cells and regulatory T cells, and increased tumour angiogenesis. Another study on IL-17 showed that it promoted infiltration of the MDSCs and Treg cells in colorectal cancer, which suppressed cytotoxic T cells and activated the STAT3 pathway, important for multiple pro-tumourous factors such as COX-2 and VEGF [114]. A study of IL-22 function in lung and breast cancer showed increased incidence of tumour metastases by suppression of NK cell function [115]. Another significant player in the tumour microenvironment is represented by regulatory T cells. They carry CD4+ CD25+ FOXP3+ phenotypes and are responsible for immune suppressive features by secretion of IL-2, IL-10, TGFβ, IL-35, and cytotoxic T-lymphocyte-associated protein 4 (CTLA-4) [116]. Multiple meta-analyses of different malignancies in humans and mice have shown negative prognosis for tumours infiltrated by Tregs, which results in weak infiltration of CD8+ lymphocytes [117,118]. Targeting of Tregs in the cancer TME could be a promising method, but a significant challenge is the high risk of development of autoimmune diseases [119,120,121].

## 8. Targeting Tumour Microenvironment Elements in Diffuse Large B Cell Lymphoma

The tumour microenvironment is a complex communication of tumour cells and surrounding tissues via small molecules, which is a challenge to target nowadays. There are multiple studies which prove the importance of TME elements such as TAMs [122], MDSCs [123], and the CCR2/CCL2 pathway [124] and their influence on the poor prognosis in patients with diffuse large B cell lymphoma.

According to the analysed literature, the most prominent targets in the TME could be TGF-β, produced by the tumour itself, TAMs, MDSCs, iCAFs and stimulation of anti-inflammatory response, and reduced infiltration of the CD8+ Tc1. Nowadays, there are multiple clinical trials trying to inhibit TGF-β activity in solid tumours, but in the case of positive results, new designed drugs could be used in the treatment of refractory lymphomas as well. One of the most promising drugs is Fresolimumab, a TGF-β1, TGF-β2, and TGF-β3 inhibitor. Phase 1 studies are now being performed or completed for melanoma, renal cell carcinoma [125], breast cancer [126], or mesothelioma [127]. One more interaction with TGF-β is performed with the use of a bifunctional fusion protein targeting TGF-β and PD-L1—Bintrafusp alfa, which is used also in many solid tumours such as non-small cell lung cancer [128], head and neck cancer [129], and HPV-positive solid tumours [130]. In treatment of lymphoma now in trials, there are specific cytotoxic T-lymphocytes resistant to the TGF-β immunosuppressive effects, which could be a new potential addition in the treatment in many scenarios, for example, CART therapy [131].

ARG1 figures in many TME elements with similar anti-Tc1 features to be a potential target for the therapy. A novel oral Arginase 1/2 inhibitor, OAT-1746, was tested in murine models with glioblastoma [132]. Arginase-1 targeting peptide vaccine is undergoing a phase 1 trial in multiple solid cancer patients [133], and dual arginase inhibitor OATD-02 has received permission for a phase 1 clinical trial in Poland for patients with advanced solid tumours [134]. Unfortunately, no clinical trials on arginase-1 inhibitors are currently in progress for lymphomas.

IL-10 could also be a potential therapy target. In vivo inhibition of it in colorectal cancer showed positive treatment results in combination with CART therapy [135]. Unfortunately, no available clinical trials targeting IL-10 in DLBCL were found.

Currently available VEGF inhibitors such as bevacizumab have shown notable positive effects in the treatment of HHV8-unrelated effusion large B-cell lymphoma (ELBCL), suppressing effusion formation and lymphoma cell growth in mouse models [136]. Studies of COX inhibitors like celecoxib have also shown better prognosis and overall survival in patients with DLBCL by blocking PGE2 and inducing cell apoptosis [137,138,139]. Interestingly, celecoxib showed negative results in patients with DLBCL, who underwent CAR-T therapy by inducing CAR-T cell apoptosis, and should be used with caution in this particular group of patients [140].

## 9. Role of the Tumour Microenvironment in Rare B Cell Lymphomas

Primary central nervous system lymphoma (PCNCL) is a rare type of non-Hodgkin lymphoma, having an incidence of only 2–3%. PCNSL follows an aggressive clinical course, with DLBCL being the most common histological type in around 90% of cases [141]. The comparative analysis between PCNSL and DLBCL suggests that PCNSL is more likely to be an immunologically deficient tumour, with a reduced number of T cells alongside M2 polarised macrophages, endothelin B receptor, HLA depletion, PD-L1, and T cell immunoglobulin, and a mucin-domain containing-3 (TIM-3) [142]. Use of flow cytometry showed the presence of both M2 and M1 macrophages. Inhibiting macrophages by CSF-1 receptor blockage led to CNS lymphoma progression, reduced T-cell infiltration and blocked rituximab efficacy, showing the important role of tumour-associated macrophages in the CNS lymphoma TME, like in the other tumours [143]. Another analysis of the CNS lymphomas microenvironment identified two major components: CD8+ T cells and both M1 and M2 macrophages. The presence of M2 was associated with a higher number of TIM-3 proteins, T cell suppressors, and greater PD-1 expression. The study also revealed TGFβR1 as a top upstream regulator of immune evasion in CNS lymphoma [144]. Analysis of the CNS lymphoma microenvironment is still complex due to the rareness of disease, and there were no large studies found with complex and broad analysis of TME in CNS lymphomas, but available studies reveal the important role of macrophages to build up a prognosis and new possibilities of targeting treatment, focused, for example, on TGF-β or PD-1 receptors.

Another challenge for clinicians is the rare CD20-negative types of DLBCL. These constitute 1–2% of B cell lymphomas, with the main presenting subtype being aggressive plasmablastic lymphoma (PL), observed in approximately 75% of cases. This subtype is frequently associated with HIV and/or Epstein–Barr virus (EBV) co-infection [145]. One study has reported significant infiltration of CD163-positive macrophages in 97% of cases, regardless of EBV status. Additionally, there was low expression of CD8+ cytotoxic markers such as granzyme B, which correlates with TAM M2 immunosuppressive abilities. EBV-positive tumours also exhibited twice larger expression of the PD1-L marker, associating EBV-positive status with a poorer prognosis [146]. Unfortunately, there are few data available regarding the tumour microenvironment (TME) of plasmablastic lymphomas. More studies focused on the TME are needed to better understand the processes involved in this subtype of B cell lymphomas and to identify potential targets for future therapy.

## 10. Conclusions

This review gives a complex insight into the tumour microenvironment and shows a rich network of mechanisms which suppress CD8+ cytotoxic T cell type 1. This could be a reason for the failure of modern DLBCL therapies such as checkpoint inhibitors, CAR-T, bispecific antibodies, and nanobodies therapies, based on the CD8+ T cell presence in the tumour niche.

Myeloid-derived suppressor cells cause immunosuppression in the TME by inhibiting cytotoxic T cells and NK cells, as well as the ability of B cells to present tumour antigens. MDSCs also stimulate proliferation of T regulatory cells and indirectly suppress immune response against malignant cells. The ability to transform into tumour-associated macrophages or neutrophils increases their negative impact.

The role of tumour-associated macrophages is strongly dependent on their polarisation and factors dominating in the TME. They may transition from anti-tumourous type M1 to the pro-tumourous M2 type and suppress cytotoxic T cells, promoting tumour proliferation and dissemination.

Tumour-associated neutrophils can directly induce tumour cytotoxicity or stimulate NK cell infiltration, but at the same time, the N2 type enhances DNA instability, increases angiogenesis, and suppresses cytotoxic T cells, which makes them pro-tumourous.

Cancer-associated fibroblasts may remodel matrix and stimulate angiogenesis to promote tumour dissemination as well as increase the presence of myeloid-derived suppressor cells.

CD8+ T cells classically count as anti-tumourous cells and are a basis of modern therapies such as immune, CART, or bispecific antibody therapies, but many studies also show pro-tumourous abilities promoting tumour growth and immunosuppression. More studies are needed to predict their role in the TME and increase the efficiency of treatment.

Novel drugs, targeting TME as TGF-β or ARG1 inhibitors, are undergoing clinical trials in cancer patients, but there is a significant lack of trials in lymphoma patients.

Targeting the angiogenesis with vascular endothelial growth factor (VEGF) inhibitors in DLBCL could be beneficial in use with standard therapy regimens.

The use of different cyclooxygenase (COX) inhibitors, especially COX-2 inhibitors, could improve patients’ prognosis, but more clinical trials should be carried out before adding them to the standard regimen schemes.

The tumour microenvironment is a complex mechanism which is ruled by tumour cells, and there is still a lack of studies of the TME in the haematological tumours such as DLBCL. More studies should be performed to isolate potential targets for evaluation of patients’ prognosis and more efficient treatment options.

## Figures and Tables

**Table 1:** Role of tumour microenvironment components.

Cell Type	Sub-Types	Major Role in TME	Function	Released Factors
TAMs	M1	Anti-tumourous	Cytotoxicity	
	M2	Pro-tumourous	Suppression of CD8+ T cells	TGF-β ARG1 IL-10 IDO
			Angiogenesis	VEGF PDGF Angioprotein 2 CXCL1 FGF2
			Promoting dissemination	MMP2 MMP9 CCL18 Cathepsin
MDSCs	PNM-MDSCs	Pro-tumourous	Suppression of CD8+ T cells	TGF-β ARG1 IL-10 IDO
	M-MDSCs	Pro-tumourous	Promoting of CD4+ Treg Suppression of NK cells	IDO1
			Suppression of CD8+ T cells and B cells	iNOS promoting PGE2
TANs	N1	Anti-tumourous	Stimulation of NK cells	IL-8
			Cytotoxicity	ROS MPO H_2_O_2_ Proteases
	N2	Pro-tumourous	DNA damage	NOS ROS
			Suppression of CD8+ T cells	ARG1
			Angiogenesis	VEGF MMP9
CAFs	mCAFs	Unspecified	Stromal matrix remodelling	MMP11 COL1A2
	iCAFs	Pro-tumourous	Promoting MDSCs	CCL2 IL-6
	vCAFs	Pro-tumourous	Angiogenesis	
	tCAFs	Pro-tumourous	Promoting tumour growth	MME CAIX TMEM158
CD8+ T cells	Tc1	Anti-tumourous	Cytotoxicity	IFN-y TNF-α Granzyme B Perforin
	Tc2	Pro-tumourous	Interactions in TME	IL-4 IL-5 IL-10
	Tc9	Pro-tumourous	Promoting tumour growth	IL-9 IL-4
	Tc17	Pro-tumourous	Promoting tumour growth Angiogenesis	IL-17
			Promoting TANs	IL-17
			Suppression of NK cells	IL-22
CD4+ Treg		Pro-tumourous	Suppression of CD8+ T cells	IL-2 IL-10 TGFb IL-35 CTLA-4

Abbreviations in the table. TAMs, tumour-associated macrophages; MDSCs, myeloid-derived suppressor cells; PMN-MDSCs, polymorphonuclear myeloid-derived suppressor cells; M-MDSCs, monocytic myeloid-derived suppressor cells; TANs, tumour-associated neutrophils; CAFs, cancer-associated fibroblasts; NK cells, natural killer cells; Treg, T regulatory cells; Tc, T cytotoxic cells; TGF-β, tumour-growing factor beta; ARG, arginase; IL, interleukin; IDO, indoleamine 2,3-dioxygenase; NOS, nitric oxide synthase; iNOS, inducible NOS; PGE2, prostaglandin E2; ROS, reactive oxygen species; MPO, myeloperoxidase; PDGF, platelet-derived growth factor; VEGF, vascular endothelial growth factor; CXCL1, chemokine (C-X-C motif) ligand 1; FGF2, basic fibroblast growth factor; MMP, matrix metalloproteinase; CCL, chemokine (C-C motif) ligand; CAIX, carbonic anhydrase IX; COL1A2, collagen 1A2; MME, membrane metallo-endopeptidase; TMEM, transmembrane protein; IFN-y, interferon gamma; TNF-α, tumour necrosis factor alpha; CTLA, cytotoxic T-lymphocyte-associated protein.

## Data Availability

Not applicable.
